# Suppression of erythropoiesis by dietary nitrate

**DOI:** 10.1096/fj.14-263004

**Published:** 2014-11-24

**Authors:** Tom Ashmore, Bernadette O. Fernandez, Colin E. Evans, Yun Huang, Cristina Branco-Price, Julian L. Griffin, Randall S. Johnson, Martin Feelisch, Andrew J. Murray

**Affiliations:** *Department of Physiology, Development, and Neuroscience and ^†^Department of Biochemistry, University of Cambridge, Cambridge, United Kingdom; and ^‡^Faculty of Medicine, Clinical and Experimental Sciences, University of Southampton, Southampton, United Kingdom

**Keywords:** kidney, oxygen sensing, hypoxia

## Abstract

In mammals, hypoxia-triggered erythropoietin release increases red blood cell mass to meet tissue oxygen demands. Using male Wistar rats, we unmask a previously unrecognized regulatory pathway of erythropoiesis involving suppressor control by the NO metabolite and ubiquitous dietary component nitrate. We find that circulating hemoglobin levels are modulated by nitrate at concentrations achievable by dietary intervention under normoxic and hypoxic conditions; a moderate dose of nitrate administered *via* the drinking water (7 mg NaNO_3_/kg body weight/d) lowered hemoglobin concentration and hematocrit after 6 d compared with nonsupplemented/NaCl-supplemented controls. The underlying mechanism is suppression of hepatic erythropoietin expression associated with the downregulation of tissue hypoxia markers, suggesting increased pO_2_. At higher nitrate doses, however, a partial reversal of this effect occurred; this was accompanied by increased renal erythropoietin expression and stabilization of hypoxia-inducible factors, likely brought about by the relative anemia. Thus, hepatic and renal hypoxia-sensing pathways act in concert to modulate hemoglobin in response to nitrate, converging at an optimal minimal hemoglobin concentration appropriate to the environmental/physiologic situation. Suppression of hepatic erythropoietin expression by nitrate may thus act to decrease blood viscosity while matching oxygen supply to demand, whereas renal oxygen sensing could act as a brake, averting a potentially detrimental fall in hematocrit.—Ashmore, T., Fernandez, B. O., Evans, C. E., Huang, Y., Branco-Price, C., Griffin, J. L., Johnson, R. S., Feelisch, M., Murray, A. J. Suppression of erythropoiesis by dietary nitrate.

Oxygen homeostasis is critical for proper organ function and survival of mammals. By means of hemoglobin (Hb)-containing red cell production (erythropoiesis) and blood flow regulation, oxygen supply is matched to tissue oxygen demand. In response to decreased cellular oxygen tensions (pO_2_), regions of the kidneys (1, 2) and the liver (3) release the hormone erythropoietin (EPO) (4), stimulating erythropoiesis and mitigating a fall in oxygen transport. Although the majority of EPO is derived from the kidneys (3), hepatic EPO production may account for one-third of total EPO under conditions of severe hypoxia (5). EPO expression in these tissues is under the control of the hypoxia-inducible factor 2 (HIF2) (6–8); this transcription factor is constitutively expressed and continuously degraded in normoxia, yet becomes stabilized under conditions of severe/prolonged hypoxia, *e.g.,* during an extended sojourn to high altitude. As such, with sufficient time to acclimate, oxygen content is maintained at sea level values in mountaineers even up to 7000 m above sea level, with increased blood Hb content compensating for decreased arterial Hb-O_2_ saturation (9).

With prolonged/severe hypoxia, however—a condition prevalent in critical illness and unavoidable at high altitude (10)—an elevated hematocrit can lead to a detrimental increase in blood viscosity (*e.g.,* in chronic obstructive pulmonary disease and Monge’s disease) (11), which may impair blood flow through the microcirculation (12). An increased hematocrit may not, therefore, substantially increase oxygen delivery when pO_2_ and oxygen saturation are low. In support of this notion, VO_2max_ remains low at high altitude, even in fully acclimatized subjects (13); of note, Tibetan natives, adapted to life at high altitude for generations, do not have the elevated hematocrit of altitude-acclimatized lowland natives (14, 15), an adaptation associated with enhanced forearm blood flow and elevated plasma nitrogen oxide levels (16).

Nitrate (NO_3_^−^), the metabolic end product of the signaling molecule NO and a ubiquitous dietary constituent, lowers the oxygen cost of exercise by improving mitochondrial efficiency (17). Nitrate may also be an alternative source of NO, particularly when oxygen availability is limited (18). A possible role of NO in the control of red blood cell development *via* effects on erythroid cells, has been suggested (19), whereas in a study using eNOS knockout mice and NOS inhibitors, endogenous NO was found to attenuate renal EPO expression in mice (20). We hypothesized that nitrate administration, *via* the diet, might limit hematocrit rises in overt hypoxia by improving the efficiency of oxygen utilization, thereby decreasing the amount of Hb needed for oxygen delivery. We set out to investigate the effects of dietary nitrate on circulating Hb in rats exposed to environmental hypoxia and found that under both hypoxic and normoxic conditions, a moderate dose of nitrate comparable to that known to enhance mitochondrial efficiency in humans (17), suppresses plasma EPO levels and lowers circulating Hb concentrations. We subsequently investigated the mechanisms underpinning these effects of nitrate and found that both liver and kidneys are involved in optimizing hematocrit in relation to environmental conditions and circulating nitrate concentrations.

## MATERIALS AND METHODS

All procedures were carried out by a license holder in accordance with UK Home Office regulations under the Animals in Scientific Procedures Act and were reviewed by the University of Cambridge Animal Welfare and Ethical Review Committee.

### Hypoxia study

Male Wistar rats (273 ± 2 g; *n* = 40) were acquired from Charles River (Margate, United Kingdom) and maintained on a standardized quality-controlled chow to normalize micronutrient levels [RM1(E)SQC; Special Diets Services, Essex, United Kingdom; 55% carbohydrate, 3% fat, 15% protein). The hypoxia/nitrate protocol was as previously described (21). After a 12 d acclimatization, animals received either 0.7 mM NaNO_3_ (nitrate group) or NaCl (control group, matched for salinity and sodium content; both ultra-pure; Sigma-Aldrich, Dorset, United Kingdom) in distilled water (*n* = 20/group). After 4 d, half of each group was transferred to hypoxia chambers at 13% O_2_ with 20 air changes/h (PFI Systems Ltd., Milton Keynes, United Kingdom; *n* = 10/group). All animals remained in these conditions, with *ad libitum* access to food and NaNO_3_ or NaCl-supplemented water, for 14 d. Animals were housed pairwise in conventional cages at controlled humidity and temperature with a normal 12 h/12 h light/dark cycle. Food and water consumptions were monitored daily, and nitrate intakes were calculated from measured nitrate contents.

### Time course and dose-response studies

To investigate the time course of the effect of nitrate supplementation, rats (267 ± 3 g; *n* = 24) were housed under normoxia and conditions otherwise identical to those above, on standardized rodent chow, with access to distilled water *ad libitum*. After 12 d, the drinking water was supplemented with 0.7 mM NaNO_3_ with measurements taken after 0, 2, 4, 6, 9, and 12 d (*n* = 4/group). To establish the dose-response relationship, male Wistar rats (269 ± 2 g; *n* = 24) were also kept under normoxic conditions and on standardized rodent chow as above, with access to distilled water *ad libitum*. After a 12 d acclimatization, rats received distilled water or water supplemented with NaNO_3_ (0.35, 0.7, 1.4 mM; *n* = 6/group) *ad libitum* for a further 18 d.

### Hemoglobin, hematocrit, and erythropoietin

On killing, blood was collected by cardiac puncture. Hb concentration was measured by an automated analyzer (HemoCue Hb 201; Ängelholm, Sweden). Hematocrit was determined by measurement of packed cell volume after centrifugation of fresh blood in heparinized capillary tubes. EPO concentrations in plasma were determined using a commercial ELISA assay (Rat EPO ELISA kit; Biorbyt, Cambridge, United Kingdom).

### Nitrate, nitrite, nitroso-compounds, and osmolality

Nitrate was quantified using a dedicated HPLC system (ENO-20; Eicom, Tokyo, Japan), using sequential ion chromatography, online reduction to nitrite using a cadmium column, and postcolumn derivatization with a modified Griess reagent, as described earlier (22). Drinking water samples were analyzed directly after dilution with ultrapure water, rodent chow after homogenization and extraction with water, and plasma samples following deproteinization with 50% methanol. The concentration of total nitroso products in plasma was quantified using triiodide-mediated reduction and gas phase chemiluminescence detection of generated NO, as described in detail elsewhere (22). Plasma osmolality was determined by freezing point depression using a micro-osmometer (Model 3320; Advanced Instruments, Norwood, MA, USA).

### Arterial oxygen saturation

*In vivo* Hb-O_2_ saturations were determined by pulse oximetry at d 0 and 10 of the dose-response study using a MouseOx System (STARR Life Sciences Corporation, Oakmont, PA, USA). Animals were allowed to acclimatize, and measurements were taken after 30 min of constant readings.

### Gene expression analysis

Total RNA was purified from crushed, frozen liver and kidney using an RNeasy Mini Kit (QIAgen, Germantown, MD, USA). RNA concentration was quantified at 260 nm using a SmartSpecPlus spectrophotometer (Bio-Rad).

For analysis of steady-state mRNA levels, relative abundance of transcripts of interest was assessed by quantitative PCR in SYBR Green FastStart Universal Master Mix (Applied Biosystems, Grand Island, NY, USA) with a StepOnePlus detection system (Applied Biosystems). QuantiTect primer assays for rat *Epo, Vegfa, Pgk1, Slc1a2, Ca9*, and *Nos2* were obtained from QIAgen. Expression levels were normalized to *Actb* using the *∆*C_T_ method and subsequently to the “normoxia/chloride” group in the hypoxia study and to the “control” group in the dose-response study to express as fold changes.

### Immunohistochemistry

Transverse paraffin-embedded kidney sections (7 *μ*m) were immunostained for HIF1*α* as described (23) with primary antibody binding detected using biotinylated rabbit anti-mouse (Dako, Carpinteria, CA, USA). Contiguous sections were also immunostained for HIF2*α* by replacing the anti-HIF1*α* antibody with an anti-HIF2*α* antibody (Novus Biologicals, Cambridge, United Kingdom). Isotype-matched IgG was used as a negative control. Images of immunostained sections were captured in a blinded fashion using a light microscope and mounted camera (M165FC and DFC310FX; Leica, Newcastle upon Tyne, United Kingdom). Positive staining was quantified as previously described (24, 25) and expressed as average percentage of transverse tissue area throughout the length of the tissue.

### Statistics

Results are expressed as mean ± sem. ANOVA was used to determine significant differences across the 4 groups of the hypoxia and dose-response studies and 6 groups of the time course study. Dunnett’s *post hoc* analyses provided significance levels between groups compared to normoxia/NaCl controls in the hypoxia study, untreated controls in the dose-response study, and d 0 controls in the time course study. A 2-tailed nonparametric Pearson correlation was used to determine Hb and EPO correlations in the dose-response and time course studies. Differences were considered significant when *P* < 0.05.

## RESULTS

### Hypoxia study

We first investigated the effects of a moderate dose of dietary nitrate on circulating Hb in normoxic and hypoxia-exposed rats.

Neither hypoxic exposure nor nitrate supplementation altered food or water intake; thus, all groups had identical calorie intakes (Table 1). Calculated dietary nitrate intakes amounted to 1 ± 0 (normoxia/NaCl), 9 ± 0 (normoxia/NaNO_3_), 1 ± 0 (hypoxia/NaCl), and 7 ± 0 (hypoxia/NaNO_3_) mg NaNO_3_/kg body weight/d. There were no significant differences in starting or final body weights between groups; however, final body weights tended to be lower in hypoxic rats, whether supplemented with nitrate or chloride (Table 1).

**TABLE 1.** Effects of 18 d supplementation with 0.7 mM nitrate on food, water, and nitrate intake, body weight, and plasma nitrogen oxide levels in normoxic and hypoxic rats (13% O_2_)

Parameter	Normoxia		Hypoxia	
	NaCl	NaNO_3_	NaCl	NaNO_3_
Intake levels				
Food (g/d)	32 ± 2	33 ± 1	35 ± 2	33 ± 1
Water (ml/d)	28 ± 1	38 ± 10	37 ± 4	32 ± 2
Nitrate (mg/kg/d)	1 ± 0	9 ± 0*	1 ± 0	7 ± 0^†^
Body weights				
Start (g)	268 ± 5	268 ± 6	276 ± 2	280 ± 4
End (g)	429 ± 8	431 ± 9	416 ± 14	412 ± 12
Plasma NO levels				
Nitrate (*µ*M)	7.1 ± 1.0	12.8 ± 1.1*	5.0 ± 0.5	9.6 ± 0.6^†^
Nitrite (*µ*M)	0.5 ± 0.1	0.4 ± 0.1	0.5 ± 0.0	0.6 ± 0.1
Nitroso-compounds (nM)	13.3 ± 0.9	14.7 ± 3.0	19.6 ± 4.9	12.6 ± 1.7

*n* = 8–10 per group. *P < 0.001 compared with normoxia/NaCl. ^†^*P* < 0.001 compared with hypoxia/NaCl.

Plasma nitrate concentrations were the same in normoxic and hypoxic NaCl-supplemented rats; however, dietary nitrate supplementation in normoxic and hypoxic rats resulted in plasma nitrate levels that were 80% (*P* < 0.001) and 35% (*P* < 0.05) greater than those in normoxia/NaCl rats (Table 1). With this dose of dietary nitrate, no differences in either circulating nitrite or nitroso-compound concentrations were observed (Table 1).

**Figure 1.**
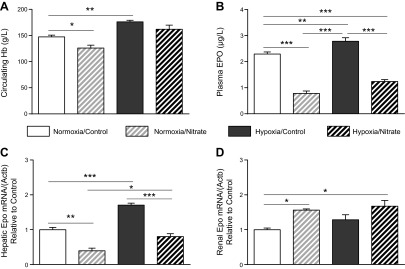
A moderate dose of dietary nitrate suppresses circulating Hb in normoxia and hypoxic rats. *A*) Circulating Hb levels in normoxic and hypoxic rats ± nitrate. *B*) Plasma EPO levels. *C*) Hepatic *Epo* mRNA levels relative to controls. *D*) Renal *Epo* mRNA levels relative to controls. *P < 0.05; **P < 0.01; ***P < 0.001.

As anticipated, circulating Hb concentrations were 20% higher in hypoxia/NaCl rats compared with normoxia/NaCl rats (*P* < 0.01). Nitrate supplementation of hypoxic rats, however, resulted in Hb concentrations that were intermediate between those of hypoxia/NaCl rats and normoxia/NaCl rats. Unexpectedly, Hb concentrations of nitrate-supplemented normoxic rats were also lower than those in normoxia/NaCl rats by 15% (*P* < 0.05; **Fig. 1*A***).

To understand whether the observed Hb-lowering effects of nitrate were associated with alterations in EPO production and/or secretion, plasma EPO levels and *Epo* expression in liver and kidney were measured. Following hypoxic exposure, plasma EPO levels reflected circulating Hb levels, being 18% higher in hypoxia/NaCl rats than in normoxia/NaCl rats (*P* < 0.01; Fig. 1*B*). Meanwhile, nitrate supplementation, in normoxic and hypoxic rats, resulted in plasma EPO levels that were 72% (*P* < 0.001) and 46% (*P* < 0.001) lower than those of normoxia/NaCl rats (Fig. 1*B*).

Across the 4 experimental groups, hepatic *Epo* expression reflected circulating EPO levels, with *Epo* mRNA being 70% higher in the livers of hypoxia/NaCl rats compared with those of normoxia/NaCl rats (*P* < 0.001; Fig. 1*C*). *Epo* mRNA levels were 71% lower in the livers of normoxia/NaNO_3_ rats compared with normoxia/NaCl rats (*P* < 0.01) and 53% lower in the same organ when comparing nitrate and chloride-supplemented groups in hypoxia (*P* < 0.001; Fig. 1*C*). In contrast, however, levels of renal *Epo* mRNA were not significantly higher in hypoxia/NaCl rats than in normoxia/NaCl rats (Fig. 1*D*), whereas *Epo* mRNA was 56% (*P* < 0.05) and 67% (*P* < 0.05) higher in the kidneys of normoxia/NaNO_3_ and hypoxia/NaNO_3_, respectively, than in those of normoxia/NaCl rats (Fig. 1*D*).

At this stage, our data suggested that the effects of nitrate were mediated *via* changes in hepatic *Epo* expression, altering circulating EPO levels. Meanwhile, the elevation of *Epo* expression by nitrate in the kidneys, which was exacerbated by environmental hypoxia, is consistent with the notion of enhanced renal hypoxia secondary to diminished oxygen delivery as a result of relative anemia.

### Time course study

Next, we investigated the time course of the effect of the same moderate level of dietary nitrate supplementation on circulating Hb levels in a separate group of rats maintained under normoxia.

**Figure 2.**
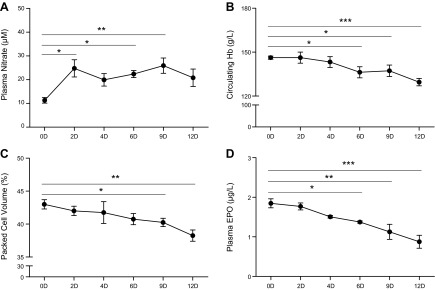
Time course of Hb suppression by dietary nitrate. *A*) Plasma nitrate levels over 12 d of nitrate supplementation. *B*) Circulating Hb levels over 12 d of nitrate supplementation. *C*) Hematocrit levels over 12 d of nitrate supplementation. *D*) Plasma EPO levels of 12 d of nitrate supplementation. *P < 0.05; **P < 0.01; ***P < 0.001.

Over 12 d of nitrate supplementation, food intake and water intake was not altered (data not shown); nitrate intake amounted to 9 mg/kg body weight/d, consistent with the earlier hypoxia study. This level of nitrate supplementation elevated plasma nitrate levels 2.2-fold to 24.8 ± 4.2 *µ*M from d 2 onward (**Fig. 2*A***), with no significant effect on plasma nitrite or nitroso levels (Supplemental Fig. 1*A, B*) and osmolality (Supplemental Fig. 1*C*). This level of nitrate supplementation resulted in a time-dependent fall in circulating Hb concentration and hematocrit (Fig. 2*B, C*), which reached significance by d 6 and 9, respectively, and remained lower for the remainder of the study. Furthermore, plasma EPO concentrations similarly fell in a time-dependent manner, reaching significance at d 6 (Fig. 2*D*).

### Dose-response study

To understand the dose dependence of the nitrate effects on circulating Hb, we carried out a dose-response study in which a further group of rats were supplemented with 0 mM (control), 0.35 mM (low dose), 0.7 mM (medium dose), or 1.4 mM (high dose) NaNO_3_ in their drinking water for 18 d. The medium dose corresponded to that used in the previous 2 studies and is essentially a repeat of the normoxia/NaNO_3_ group in the hypoxia study. Nitrate intakes amounted to 1.2 ± 0.1, 4.2 ± 0.1, 7.0 ± 0.4, and 12.1 ± 0.6 mg NaNO_3_/kg body weight/d in control, low dose, medium dose, and high dose groups, respectively. No dose of NaNO_3_ had any effect on growth, food intake, or water intake (Table 2).

**TABLE 2.** Effects of 18 d supplementation with low (0.35 mM), medium (0.7 mM), and high (1.4 mM) doses of dietary nitrate on food, water, and nitrate intake, body weight, and plasma NO levels in rats

Parameter	Control	Low	Medium	High
Intake levels				
Food intake (g/d)	32 ± 1	32 ± 1	31 ± 0	32 ± 1
Water intake (ml/d)	41 ± 5	35 ± 3	37 ± 4	32 ± 2
Nitrate intake (mg/kg/d)	1 ± 0	4 ± 0*	7 ± 0*	12 ± 1*
Body weights				
Start (g)	263 ± 2	271 ± 4	262 ± 4	281 ± 3
End (g)	431 ± 18	434 ± 17	405 ± 9	414 ± 22

*n* = 5–6 per group. *P < 0.001 compared with control rats.

**Figure 3.**
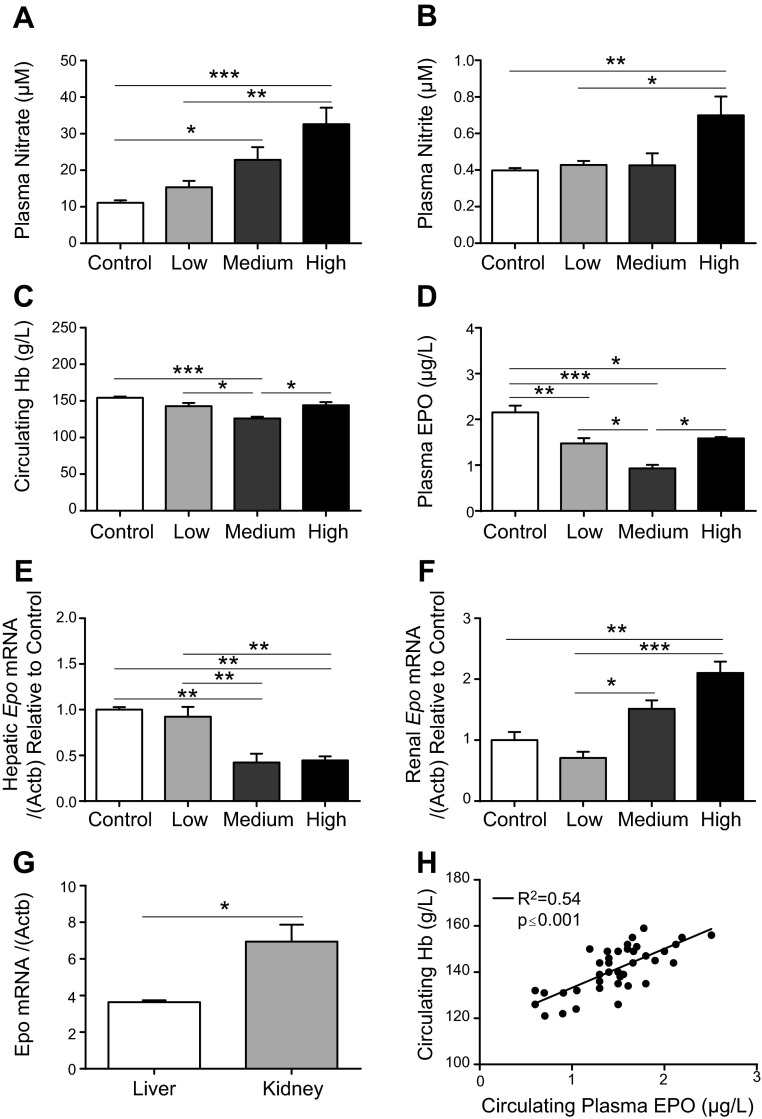
Dose-dependent modulation of circulating Hb by dietary nitrate supplementation. *A*) Plasma nitrate levels in controls and after 18 d supplementation with low (0.35 mM), medium (0.7 mM), and high (1.4 mM) doses of dietary nitrate. *B*) Plasma nitrite levels. *C*) Circulating Hb levels. *D*) Plasma EPO levels. *E*) Hepatic *Epo* mRNA levels relative to controls. *F*) Renal *Epo* mRNA levels relative to controls. *G*) Hepatic *vs.* renal Epo expression in control rats. *H*) Correlations between circulating hemoglobin levels and plasma EPO levels and circulating Hb levels and plasma nitrate concentration across time course and dose-response studies. *P < 0.05; **P < 0.01; ***P < 0.001.

Circulating plasma nitrate levels were dose-dependently increased on nitrate supplementation (**Fig. 3*A***). In the medium and high dose groups, nitrate supplementation resulted in circulating nitrate levels that were 2.1-fold (*P* < 0.05) and 2.9-fold (*P* < 0.001) higher than in controls. Plasma nitrite was not elevated in the low or medium dose groups, but was 1.8-fold (*P* < 0.01) higher in the high dose group compared with controls (Fig. 3*B*). Finally, circulating nitroso species were not significantly different across the 4 groups, although they trended upward as dose increased (Supplemental Fig. 2*A*). No dose of nitrate altered plasma osmolality (Supplemental Fig. 2*B*) or serum total protein concentration (Supplemental Fig. 2*C*).

In agreement with the hypoxia study, the medium dose of nitrate was associated with 20% lower Hb concentration, compared with controls, whereas the low dose resulted in a Hb concentration that was lower than controls but did not reach significance (Fig. 3*C*). Interestingly, at the high dose of nitrate supplementation, the anemic response was partially reversed (Fig. 3*C*). Arterial Hb-O_2_ saturation was not affected by nitrate (Supplemental Fig. 2*D*).

Plasma EPO levels reflected circulating Hb concentrations in these groups, being 24% (*P* < 0.01), 52% (*P* < 0.001), and 18% (*P* < 0.05) lower in the low, medium, and high dose groups, respectively, compared with controls (Fig. 3*D*).

At the tissue level, hepatic *Epo* mRNA levels were the same in the low dose group as in controls, but were 58% and 55% lower in the medium and high dose rats, respectively, compared with controls (*P* < 0.01; Fig. 3*E*). Meanwhile, levels of renal *Epo* mRNA in the low and medium dose groups were not significantly different from controls, but were 110% higher with high nitrate compared with controls (*P* < 0.01; Fig. 3*F*). These findings are consistent with the notion that nitrate lowers hepatic *Epo* expression, lowering EPO plasma levels, whereas renal hypoxia is exacerbated by excess anemia, resulting in elevated *Epo* expression at higher levels of nitrate.

In controls, *Epo* mRNA levels were 2-fold greater in whole kidney homogenates compared with liver (Fig. 3*G*). Across both the time course and dose-response studies, circulating Hb concentrations correlated positively with plasma EPO (*r*^2^ = 0.54; *P* < 0.001; Fig. 3*H*).

### HIF signaling

To further elucidate the mechanisms underlying the dose-dependent effects of nitrate on circulating Hb and the possible role of tissue hypoxia, we investigated HIF1*α* and HIF2α expression in the kidneys of the rats in the dose-response study, as well as HIF1 target gene expression in the liver and kidneys of these rats.

**Figure 4.**
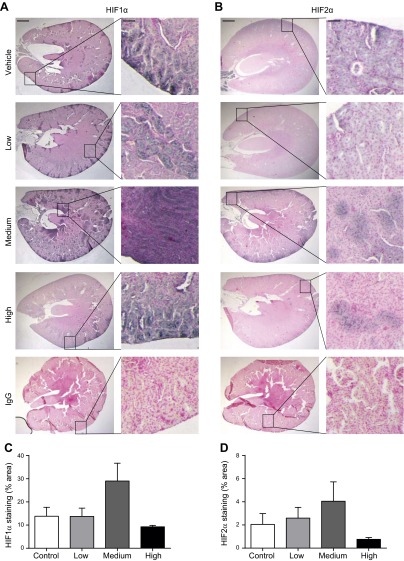
Localization and quantification of HIF1*α* and HIF2*α* in kidneys of nitrate-treated rats. *A*) HIF1*α* and *B*) HIF2*α* staining (black) with nucleated cells (red) in the kidney of treated rats and controls. Images are representative (>3 kidney sections/rat, *n* = 4–6/group). Scale bars are 2 mm and 50 *μ*m in left and right columns, respectively. *C*) Quantification of HIF1*α* and (*D*) HIF2*α* staining in the kidney of treated rats and controls (*n* = 4–6/group). Increases in HIF1*α* and HIF2*α* staining between controls and rats receiving the medium treatment dose did not reach statistical significance (*P* = 0.066 and *P* = 0.29, respectively).

Renal HIF1*α* and HIF2*α* staining (black) was detected in control and nitrate-treated rats (**Fig. 4**). Positive staining for HIF1*α* and HIF2*α* was observed in nucleated cell-populated regions and appeared to be associated with cell nuclei (red) (Fig. 4*A, B*). Contiguous sections exposed to isotype-matched IgG did not stain positively (Fig. 4*A, B*). HIF1*α* staining also appeared to be stronger than HIF2*α* staining in all groups (Fig. 4*A, B*, quantified in Fig. 4*C, D*). There were no differences in HIF1*α* or HIF2*α* staining between controls and rats receiving either low or high treatment doses (Fig. 4*C, D*). Although HIF1*α* and HIF2*α* staining was 2-fold greater in rats receiving the medium dose compared with controls, these differences did not reach statistical significance (HIF1*α*: 29 ± 8 *vs*. 14 ± 4% in controls, *P* = 0.066; Fig. 4*C*; HIF2*α*: 4 ± 2 *vs*. 2 ± 1% in controls, *P* = 0.29; Fig. 4*D*).

**Figure 5.**
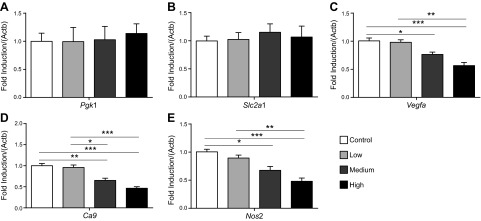
HIF1 target gene expression in livers of rats treated with dietary nitrate. *A*) Pgk1, (*B*) Slc2a1, (*C*) Vegfa, (*D*) Ca9, and (*E*) Nos2 levels in livers of control rats and after 18 d supplementation with low (0.35 mM), medium (0.7 mM), and high (1.4 mM) doses of dietary nitrate. Gene expression (normalized to Actb) is expressed relative to the control group. *P < 0.05; **P < 0.01; ***P < 0.001.

**Figure 6.**
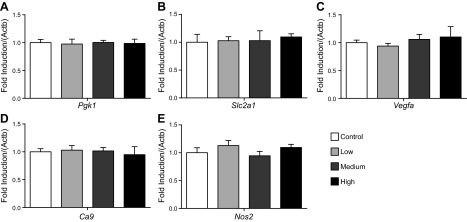
HIF1 target gene expression in kidneys of rats treated with dietary nitrate. *A*) Pgk1, (*B*) Slc2a1, (*C*) Vegfa, (*D*) Ca9, and (*E*) Nos2 levels in kidneys of control rats and after 18 d supplementation with low (0.35 mM), medium (0.7 mM), and high (1.4 mM) doses of dietary nitrate. Gene expression (normalized to Actb) is expressed relative to the control group.

In the liver, levels of *Pgk1* and *Slc2a1* (HIF1 targets) were the same in all groups; however, levels of *Vegfa, Ca9*, and *Nos2* (HIF1 targets) were all dose-dependently decreased by nitrate supplementation (**Fig. 5**), further supporting an effect of nitrate on suppression of HIF signaling in this organ. By contrast, in whole kidney homogenates, no changes were observed in the levels of any HIF1 targets measured in response to any dose of nitrate supplementation (**Fig. 6**).

## DISCUSSION

Here we report that suppressor control by the NO metabolite and ubiquitous dietary component, nitrate, on the production of hepatic erythropoietin can act to lower circulating Hb. As expected, environmental hypoxia increased circulating Hb in rats; however, moderate nitrate supplementation largely prevented this rise. Moreover, nitrate decreased Hb in normoxic rats. This result, together with an observed inverse association between nitrate and Hb in human blood (13), suggests that nitrate impacts on erythropoiesis even in normoxia.

NaNO_3_ caused a time-dependent decrease in circulating Hb, EPO, and hematocrit, reaching significance after d 6. This time course of events is distinct from nitrate’s more acute effects on mitochondrial respiration during an exercise challenge (17) or ischemic insult and consistent with a suppression of EPO-mediated erythropoiesis. Interestingly, however, the intermediate nitrate dose tested caused the maximal fall in Hb, with the anemic response partially reversed on supplementation with 1.4 mM NaNO_3_. No level of nitrate supplementation tested changed osmolality or plasma protein concentration. Changes in circulating EPO mirrored those of Hb, suggesting an effect on tissue oxygen sensing.

In agreement with this notion, above a threshold plasma concentration of ∼20 *µ*M, nitrate mediated a suppression of hepatic *Epo* expression with a concomitant fall in HIF1 target expression, suggesting increased hepatic pO_2_, either due to a suppression of tissue oxygen demand, perhaps *via* mitochondrial action (17), or an improvement in oxygen delivery (16). The partial reversal of this effect at higher levels of nitrate was associated with increased renal *Epo* expression, possibly as a result of renal hypoxia due to the relative anemia brought about by nitrate’s effects on hepatic *Epo*. Indeed, acute anemia has been shown to stabilize renal HIF1 and HIF2 in mice, whilst renal EPO production increases exponentially on hemodilution (26). In agreement, at the medium dose of nitrate used in the present study, at which Hb concentrations were lowest, we observed greater expression of HIF1*α* and HIF2*α* in the kidney; however, renal HIF levels were normalized in rats treated with the higher dose of nitrate, when the relative anemia was alleviated. Although we did not measure a change in renal HIF1 target gene expression, the localization of HIF1 and HIF2 to distinct regions of the kidney indicates the limitations associated with making these measurements in whole tissue homogenates. Given the elevation in plasma nitrite levels observed at the higher dose of nitrate supplementation, it is possible that the rise in Hb at this dose was accompanied by enhanced local formation of methemoglobin (MetHb) *via* an interaction between Hb and nitrite; however, plasma concentrations of nitrate and nitrite, even at this highest dose, remain below those required to induce methemoglobinemia. Although it is a possible limitation of this study that we did not measure MetHb, or for that matter carboxyhemoglobin, it is unlikely that compromised oxygen delivery secondary to focal Hb oxidation would be the sole contributor of this reactivation of EPO production, because at the medium dose of nitrate supplementation, which was not associated with elevated plasma nitrite, renal *Epo* expression and HIF2 were already increased.

**Figure 7.**
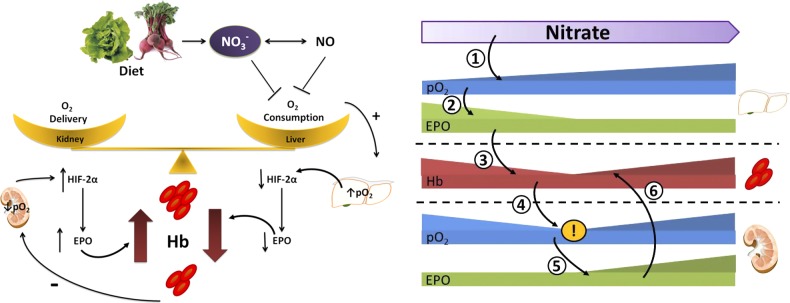
Schematic outlining the potential mechanism underlying dose-dependent effects of dietary nitrate on renal and hepatic erythropoietin expression (*1*). Nitrate supplementation enhances hepatic pO2 (*2*), suppressing Epo expression and thereby (*3*) lowering circulating Hb (*4*). At higher doses of nitrate, the relative anemia resulting from hepatic effects results in renal hypoxia (*5*), enhancing renal Epo expression and (*6*) bringing about a partial reversal in the suppression of circulating Hb levels.

The dose-response relationship indicates that this phenomenon is relevant to fluctuations within the physiologic nitrate concentration range, which in humans varies with dietary intake (primarily from green leafy vegetables) and physical activity. Several factors other than O_2_, *e.g.,* cytokines, hormones including angiotensin-II (4), and oxidative stress (27), are known to regulate EPO and Hb levels. Indeed it has been suggested, following studies using eNOS knockout mice and NOS inhibitors, that endogenous NO production attenuates HIF stabilization, and secondarily, EPO transcription (20). The novelty of our findings lies in demonstrating the effects of an endogenous metabolite and ubiquitous dietary component, recognized as a modulator of oxygen delivery and oxygen utilization by tissues, on pathways regulating oxygen-carrying capacity within the range of its normal physiologic concentrations. The predominant effect of nitrate would appear to be a suppression of hepatic EPO production, and although this is thought to represent a relatively small proportion of total EPO (∼10% of total EPO production) (3), this proportion might increase to ∼33% during prolonged/severe hypoxia (5). As such, hepatic EPO production would appear to be particularly susceptible to fluctuations in tissue oxygenation due to external factors. Consequently, hepatic EPO production appears to mediate blood oxygen-carrying capacity such that it continues to meet tissue oxygen demand without excessively raising blood viscosity, which might otherwise impair microcirculatory flow and hence tissue oxygen delivery. The renal brake on this mechanism, as demonstrated here by higher doses of nitrate supplementation, is likely to be mediated by hypoxia-sensing pathways that are activated following relative anemia, thereby averting a detrimental fall in oxygen-carrying capacity. Although our findings do not rule out nitrate-induced effects on hematocrit that might occur downstream of EPO production (*e.g.,* on EPO sensing, erythropoiesis, or red cell lifespan) across our studies, we found a correlation between plasma EPO and blood [Hb]. As such, hepatic and renal mechanisms appear to work in concert to optimize tissue oxygen delivery (**Fig. 7**).

We and others have shown that the same dose of nitrate (0.7 mmol/L) used here modulates oxygen consumption across a range of tissues in rodents (21, 28) and humans (17, 18), and thus it is seems intuitive that such a signal might also interact with mechanisms that determine oxygen delivery to the tissues in order that supply and demand mismatches do not occur. Hematocrit is an important determinant of aerobic work capacity, whereas anemia is an independent prognostic factor in several chronic illnesses, underlining the importance of such regulation. The regulatory mechanism we report here may also relate to the adaptation to chronic environmental hypoxia in Tibetan highlanders (16), who exhibit high circulating nitrate and low Hb concentrations. Moreover, there has been a recent resurgence of interest in the mechanisms of hepatic EPO production (29) stimulated by the suggestion that hepatic EPO synthesis could replace the need for recombinant EPO administration in patients with anemia secondary to chronic kidney disease (30). Indeed, during fetal life, the liver rather than the kidneys is the major source of EPO, and as such, the relevance of our findings to fetal health would be of great interest (31). Finally, our findings offer potential therapeutic avenues for dietary intervention in polycythemia and other conditions warranting a reduction in hematocrit (11).

A major strength of our study was the use of a standardized quality-controlled diet and deionized water, which allowed us to acutely manipulate micronutrient concentrations in rats while accurately monitoring nitrate intake. Nitrate concentrations vary in tap water and feed, yet the precise titration of nitrate intake achieved here, without restricting food or water intake, allowed us to study an effective dose response that resulted in plasma nitrate levels that were tightly matched within and distinct between groups. Moreover, no interventions affected food (and thus calorie) intake, which is critical because caloric restriction itself has been shown to suppress EPO production (32, 33). The use of rats, rather than mice, as a model organism was deliberate as, unlike mice, NO production rates (34) and circulating nitrate/nitrite concentrations (35) are very similar to those of humans. The intermediate nitrate dose used here matches that used in human studies (17) and can be readily achieved in humans *via* a slight modification of the diet. By using rats rather than mice, however, we were unable to capitalize on genetic models to further study these mechanisms. Another strength of this investigation is the consistency in findings across 3 independent studies and a wide range of techniques, which has allowed us to present a comprehensive, integrated mechanism for the control of hemoglobin by nitrate. A possible weakness of the study, however, is that our interventions were relatively short in duration, and longer-term studies of this effect, in rats and in humans, would be of great interest. Moreover, the implications of these findings for the delivery of oxygen to other tissues, during rest or times of increased demand, *e.g.,* acutely during an exercise challenge or chronically during pregnancy, deserve further attention.

In conclusion, at levels of intake that correspond to those readily achievable *via* the diet, nitrate modulates EPO production, suppressing hepatic *Epo* expression. At higher doses of dietary nitrate, a partial reversal of this effect occurs *via* increased renal *Epo* expression. Thus, hepatic and renal hypoxia-sensing pathways act in concert to modulate circulating Hb in response to nitrate supplementation (and presumably other modes of variation in circulating nitrate concentrations arising from differences in bodily NO production). Suppression of hepatic *Epo* expression acts to decrease blood viscosity while matching oxygen supply to tissue oxygen demand, whereas renal oxygen sensing acts as a brake, averting a potentially detrimental fall in hematocrit.

## Supplementary Material

Supplemental Data

## Abbreviations

EPO/Epo: erythropoietin
Hb: hemoglobin
Hb-CO: carboxyhemoglobin
HIF: hypoxia-inducible factor
MetHb: methemoglobin
NO_3_^−^: nitrate
pO_2_: oxygen tension
