# Supplementary material for: Suppression of erythropoiesis by dietary nitrate
Source: FASEB J. 2014 Nov 24;29(3):1102–12. doi: 10.1096/fj.14-263004 (PMC4422362; doi:10.1096/fj.14-263004)
Supplement: Supplemental Data [file supp_29_3_1102__index.html]

Suppression of erythropoiesis by dietary nitrate — Supplemental Data 

# Suppression of erythropoiesis by dietary nitrate

## Supplemental Data

**Files in this Data Supplement:**

- Supplemental Data
- Supplemental Data
- Supplemental Data
