# Supplementary material for: Suppression of erythropoiesis by dietary nitrate
Source: FASEB J. 2014 Nov 24;29(3):1102–12. doi: 10.1096/fj.14-263004 (PMC4422362; doi:10.1096/fj.14-263004)
Supplement: Supplemental Data [file supp_fj.14-263004_Supplemental_Figure1.pdf]

Data Supplement For:

Suppression of Erythropoiesis by Dietary Nitrate in Rats

Tom Ashmore,[1,2] Bernadette O. Fernandez,[3] Colin E. Evans,[1] Yun Huang,[1] Cristina Branco-Price,[1] Julian L. Griffin,[2] Randall S. Johnson,[1] Martin Feelisch*[3] and Andrew J. Murray*[1]

[1]Department of Physiology, Development & Neuroscience and [2]Department of Biochemistry, University of Cambridge, UK

[3]Faculty of Medicine, Clinical & Experimental Sciences, University of Southampton, UK

Running Title: Suppression of erythropoiesis by nitrate

For correspondence: Dr Andrew Murray
Department of Physiology, Development & Neuroscience
University of Cambridge
Downing Street
Cambridge
CB2 3EG
United Kingdom
Telephone: (+44 1223) 333863
Facsimile: (+44 870) 135 7474
Email: ajm267@cam.ac.uk

* AJM and MF are joint senior authors.

Supplementary Figures

Figure S1 **Time-course of effects of dietary nitrate supplementation. A)** Plasma nitrite levels, **B)** plasma nitroso-compound levels and **C)** plasma osmolality over 12 days of nitrate supplementation.

Figure S2 **Dose-dependent changes due to dietary nitrate supplementation. A)** Plasma nitroso-compound levels in controls and after 18 days supplementation with low (0.35 mM), medium (0.7 mM) and high (1.4 mM) doses of dietary nitrate; **B)** Plasma osmolality; **C)** Total serum protein; **D)** Arterial Hb-O_2 saturation.
