# Supplementary figures and images for: Suppression of erythropoiesis by dietary nitrate
Source: FASEB J. 2014 Nov 24;29(3):1102–12. doi: 10.1096/fj.14-263004 (PMC4422362; doi:10.1096/fj.14-263004)

Figure S1

A

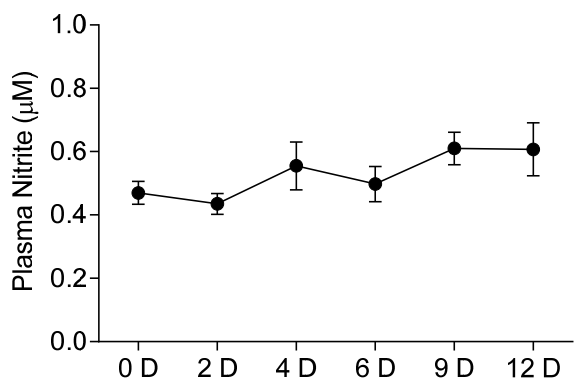

B

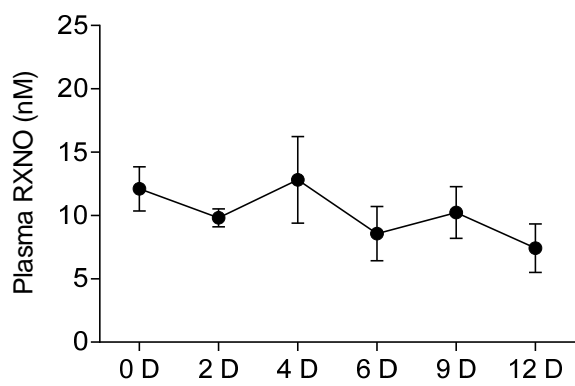

C

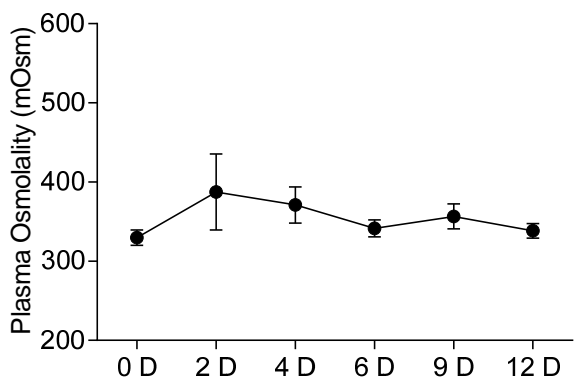

Supplement: Supplemental Data [file supp_fj.14-263004_Supplemental_Figure2.pdf]

Figure S2

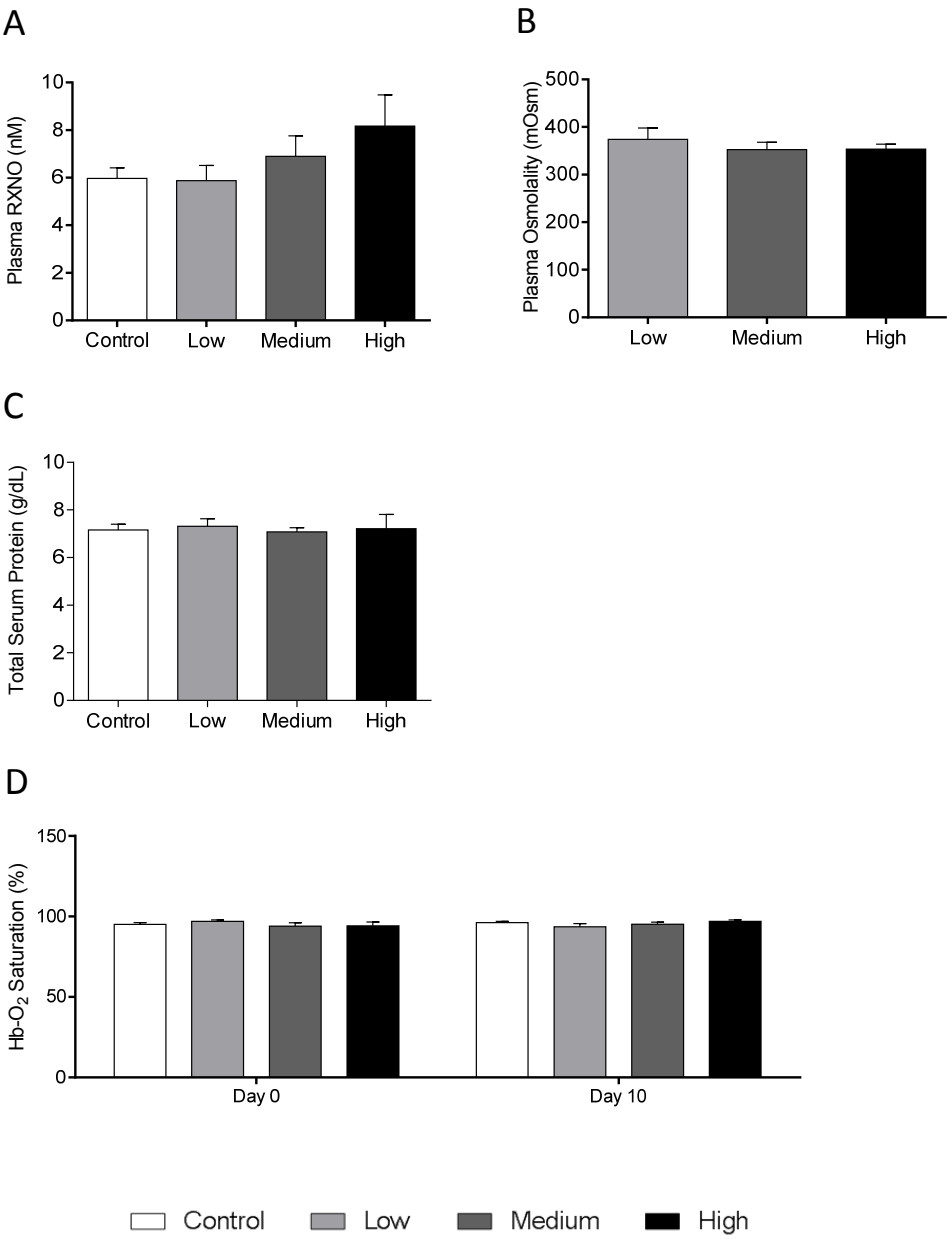

Supplement: Supplemental Data [file supp_fj.14-263004_Supplemental_Figure3.pdf]
